# Clinical and Echocardiographic Correlates of Iron Status in Chronic Heart Failure Patients: A Cross-Sectional Descriptive Study

**DOI:** 10.7759/cureus.39998

**Published:** 2023-06-05

**Authors:** Uchenna M Amaechi, Ewelukwa Chukwudum, Henry O Aiwuyo, Nosakhare Ilerhunmwuwa, John O Osarenkhoe, Anthony G Kweki, Okoke Eseoghene Onuwaje, Julius O Obilahi, Godstime I Irabor, Sheila Attuquayefio

**Affiliations:** 1 Internal Medicine, Lagos University Teaching Hospital, Lagos, NGA; 2 General Practice, Love Your Menses, Inc., Boston, USA; 3 Internal Medicine, Brookdale University Hospital and Medical Center, New York, USA; 4 Internal Medicine, Brookdale Hospital Medical Center, New York, USA; 5 Medicine and Surgery, Igbinedion University Teaching Hospital, Benin City, NGA; 6 Internal Medicine and Cardiology, Colchester Hospital, East Suffolk and North Essex NHS Foundation Trust, Colchester, GBR; 7 Cardiology and Acute Medicine, Royal Blackburn Teaching Hospital, Blackburn, GBR; 8 General Medicine, Maidstone General Hospital, Kent, GBR; 9 Pathology, Saba University School of Medicine, Saba, NLD; 10 Internal Medicine, Korle Bu Teaching Hospital, Accra, GHA

**Keywords:** correlation, cross-sectional descriptive study, chronic heart failure, clinical parameters, iron status

## Abstract

Background: Chronic heart failure (HF) is one of the conditions commonly seen in the medical outpatient departments, and iron deficiency (ID) has been reported as the commonest nutritional deficiency in these patients. The presence of ID may interfere with the clinical parameters of chronic HF. The relationship between iron status and chronic HF needs more attention and should be given more consideration in the evaluation of patients with chronic HF.

Aim: The aim of the study was to determine the relationship, if any, between iron status and clinical/echocardiographic variables in chronic HF.

Methods and materials: A cross-sectional descriptive study was carried out at the Lagos University Teaching Hospital (LUTH), Nigeria, where 88 patients with chronic HF were recruited to participate in this study. The participants underwent clinical and laboratory assessments. Iron status was assessed with full blood count parameters; serum ferritin and transferrin saturation (Tsat) and its relationship with clinical parameters among these participants were also studied.

Results: No correlations existed between the duration of chronic HF and iron status when compared using Tsat. However, a significant weak negative correlation was observed between the duration of HF and the serum ferritin levels. The clinical characteristics of the HF participants with and without ID were compared. There was no significant difference in the frequency of prior hospitalization in both groups. However, a higher proportion of participants with severe HF (New York Heart Association (NYHA) classes III/IV) (n = 14; 46.7%) were iron-deficient compared to those with moderate chronic HF (NYHA II) (n = 11; 36.7%). This relationship was statistically significant. Left ventricular ejection fraction (LVEF) was similar in the iron-deficient and iron-replete groups (using serum ferritin or Tsat) both when compared as means and when compared after categorizing LVEF as HF with preserved ejection fraction (HFpEF) vs HF with reduced ejection fraction (HFrEF). There was no statistically significant correlation between the severity of ID and LVEF.

Conclusion: A spectrum of clinical changes occurs in patients with chronic HF. ID can make these changes more profound and the condition less amenable to standard HF treatments. These patients may therefore benefit from further evaluation for this nutritional deficiency. Laboratory measurements including Tsat and serum ferritin may help in further assessment of select patients with worse and/or non-responsive clinical parameters.

## Introduction

Heart failure (HF) has a myriad of causes, and its management may be quite challenging even to an astute clinician. Among the general adult population, the frequency of HF is said to be 1-2% [1]. It is said to constitute 2.21% of out-patients, 30% of hospital admissions in cardiovascular units in Africa, and 3-7% of general medicine admissions in Sub-Saharan Africa [2,3]. These patterns are similar to what has been documented by studies conducted in Nigeria [4-6]. 

HF is a clinical syndrome characterized by typical symptoms including breathlessness, ankle swelling, and fatigue that may be accompanied by signs such as elevated jugular venous pressure, pulmonary crackles, and peripheral oedema and caused by a structural and/or functional cardiac abnormality, resulting in a reduced cardiac output and/or elevated intracardiac pressures at rest or during stress [7]. When haemoglobin (Hb), which is the major transporter of oxygen in humans, occurs at low levels, it is referred to as anaemia. It has been recorded in many studies that anaemia occurs as a comorbidity of advanced HF and also acts as an independent predictor of poor outcomes in patients. Among chronic HF patients, the frequency of anaemia was found to be 10-50%. The frequency also increased as the disease severity worsened [7,8]. As stated by the Outcomes of a Prospective Trial of Intravenous Milrinone for Exacerbations of Chronic Heart Failure (OPTIME-CHF) trial, iron deficiency (ID) makes up 21% of all cases of anaemia among HF patients [9]. 

Iron is one of the first five important micronutrients along with zinc, folate, iodine, and vitamin A [10]. Iron is particularly important in the synthesis of the haem portion of Hb used by red blood cells for the transport of oxygen to tissues. Myoglobin in muscles also contains iron. Iron also functions in several metabolic reactions as a catalyst and co-factor, as it alternates between ferric and ferrous forms; the two forms exist in the body [11,12]. Dietary sources of iron include lean meat, seafood, and poultry for haem iron and nuts, beans, and vegetables for non-haem iron. Also in certain countries like Canada and the United States, wheat and other flours are fortified with iron, which also serves as a source of dietary iron [13]. Iron, when absorbed by mucosal intestinal cells into the bloodstream through several transport mechanisms, is found in two pools: utilized pools and stored pools [14-16]. Transferrin binds to the circulating iron, which is delivered to cells to become intracellular iron. This comprises the utilized pools. Within reticuloendothelial cells, the stored pool in the form of ferritin shells is found in the cells of the liver, bone marrow, and spleen [17]. 

The human body is highly efficient in conserving its iron stores although minimal losses occur daily from the shedding of intestinal epithelial cells, skin, and appendage cells. However, considerable losses can also occur from monthly blood loss in females from menstruation [18,19]. Iron is very important in normal metabolic functioning as its deficiency has gross clinical consequences, which go beyond impairment of erythropoiesis to impaired oxidative metabolism, cellular immune mechanisms, and energetics [11,20]. 

ID may co-exist in patients with chronic HF, but little has been done to determine its presence in Black patients with chronic HF in the Sub-Saharan environment. Also, there is a paucity of data showing the relationship between the clinical parameters identified in chronic HF and the iron status of these patients in our African setting. This study evaluated the iron status in the participants and determined the clinical and echocardiographic correlates of ID in chronic HF, where present. The information from this study may influence management protocols, which would potentially include evaluation of iron status in chronic HF patients and iron treatment in those with ID to improve outcomes in the Sub-Saharan environment.

## Materials and methods

This was a cross-sectional descriptive study carried out at the Lagos University Teaching Hospital (LUTH), a tertiary hospital located in Idi-Araba, Surulere, Lagos State, Nigeria. Known adult patients visiting cardiology outpatient clinics with chronic HF were recruited for this study.

Applying the formula for cross-sectional studies for populations less than 10,000 (n* *= (*Z*^2^*pq*)/*d*^2^) [21] while using a prevalence rate of 26% of ID in chronic HF, based on serum ferritin and/or transferrin saturation (Tsat) [22], a sample size of 95 was obtained with a margin error of 10% and confidence interval of 95%. A convenient sampling technique was used to choose the patients who constituted the study population.

Interviewer-administered questionnaires were used to obtain patients' sociodemographic data and anthropometric measurements, including weight and height. Blood pressure data were also obtained from these patients. Haematological parameters assessed among the participants included Hb concentration, serum ferritin, serum transferrin, and peripheral blood film features.

Ethical permission was granted by the Lagos University Teaching Hospital Health Research Ethics Committee (approval number NHREC:19/12/2008a).

Data generated from the clinical, biochemical, and haematological parameters were analyzed using the IBM SPSS Statistics for Windows, Version 20 (Released 2011; IBM Corp., Armonk, New York, United States). Normally distributed numerical data were presented as means and standard deviations, while nonparametric data were expressed as medians and interquartile ranges. Categorical variables were presented as proportions. Means were compared using Student's t-test. Chi-square statistics were used to compare proportions between groups. Regression analysis was used to demonstrate the association between iron status and clinical variables. P values less than 0.05 were considered significant.

Categorical variables were compared using the chi-square test or Fisher’s exact test when cell counts were less than 5. The medians of variables with a skewed distribution were compared using the Mann-Whitney U test. Correlation analysis was performed to demonstrate the association between iron status and clinical parameters. Pearson’s correlation was performed for normally distributed variables, while Spearman’s rank correlation was employed for variables that did not meet normality assumptions. Forward stepwise multiple linear regression analyses were performed to determine the clinical and laboratory parameters that were independently associated with Tsat and serum ferritin levels among the study population. Forward stepwise binary logistic regression analysis was also performed to determine the clinical parameters that were independently associated with iron status.

## Results

Sociodemographic characteristics

The patients recruited for this study were between 25 and 78 years of age. On average, they were 53.99 years old. More than half, or 55.7%, of the people taking part in the study were between the ages of 45 and 64, which is considered middle-aged. There were slightly more women than men, with 47 women and 41 men. The ratio of men to women was 0.9 to 1. Most of the participants in the study had a tertiary level of education (55.7%), 26.1% had a secondary level of education, and some had only finished primary school (15.9%). Only a few (2.3%) had never gone to school. The average body mass index (BMI) was 27.61±6.42 (see Table 1).

**Table 1:** Sociodemographic characteristics of the study population (N = 88)

Parameter	Values
Overall mean age in years, mean±SD	53.93±12.52
Male	54.80 ±10.38
Female	53.15±14.19
Gender, n (%)	
Male	41 (46.59)
Female	47 (53.41)
Level of education, n (%)	
No formal education	2 (2.3)
Primary	14 (15.9)
Secondary	23 (26.1)
Tertiary	49 (55.7)
Previous hospitalizations, mean±SD	0.85±0.89
Body mass index, kg/m^2^	27.61±6.42

**Figure 1:**
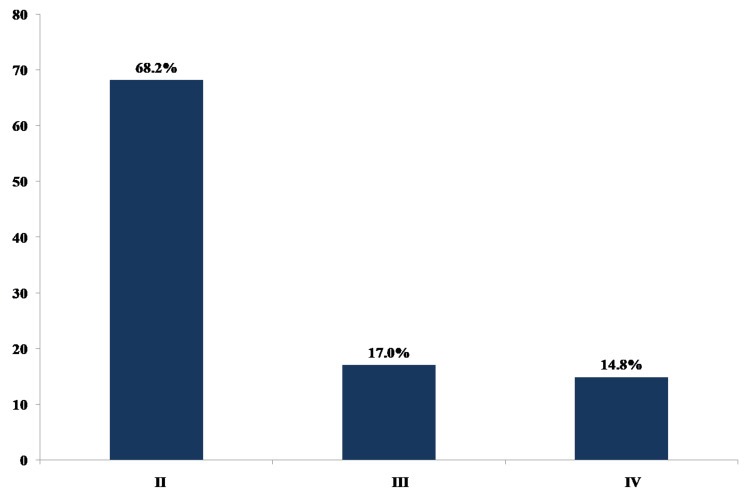
The New York Heart Association (NYHA) classification of heart failure Median NYHA class (IQR) = 2 (2-3)

The New York Heart Association (NYHA) classification of HF is shown in Figure 1.

The prevalence of anaemia and ID is shown in Table 2. Anaemia was observed in 44.3% of the total population, 58.5% in men and 31.9% in women; 30% of the total population had ID.

**Table 2:** Prevalence of anaemia and iron deficiency N = 88. iron deficient^1^: based on serum ferritin cut-off <100 mcg/L; iron deficient^2^: based on Tsat cut-off of <20% with serum ferritin 100-299 mcg/L HFpEF: heart failure with preserved ejection fraction; HFmrEF: heart failure with mid-range ejection fraction; HFrEF: heart failure with reduced ejection fraction; Tsat: transferrin saturation

Parameter	n (%)
Anaemia	39 (44.3)
Male	24 (58.5)
Female	15 (31.9)
Iron deficiency (all)	30 (34.0)
Iron deficiency^1^	15 (17.0)
Iron deficiency^2^	15 (17.0)
HFpEF	38 (43.2)
HFmrEF	18 (20.5)
HFrEF	32 (36.8)
Peripheral blood film	
Normochromic normocytic	72 (81.8)
Hypochromic microcytic	11 (12.5)
Macrocytic	5 (5.7)

Correlation between selected clinical parameters and iron status

There was a statistically significant weak inverse correlation between serum ferritin levels and the duration of HF (r* *= -0.206, p = 0.03). No correlation was observed between serum ferritin levels and age (p* *= 0.10), BMI (p* *= 0.45), frequency of hospitalizations (p* *= 0.42), pulse rate (p* *= 0.10), systolic blood pressure (p* *= 0.33), diastolic blood pressure (p* *= 0.39) and left ventricular ejection fraction (LVEF) (r* *= 0.35). There was also no association between Tsat and any of the selected clinical parameters with one exception. A weak signal (p* *= 0.05) was seen between Tsat and pulse rate (see Table 3).

**Table 3:** Relationship between serum ferritin, Tsat, and selected clinical parameters r: Pearson’s correlation coefficient; BMI: body mass index; Tsat: transferrin saturation; PR: pulse rate; SBP: systolic blood pressure; DBP: diastolic blood pressure.

Parameter	Tsat		Serum ferritin	
	r	p value	r	p value
Age	-0.030	0.39	0.139	0.10
BMI	-0.011	0.46	0.130	0.45
Duration of HF	-0.180	0.43	-0.206	0.03
Frequency of hospitalizations	-0.092	0.20	0.127	0.42
PR	-0.182	0.05	-0.094	0.10
SBP	-0.003	0.49	0.032	0.33
DBP	-0.059	0.29	-0.066	0.39

For this analysis, the population was grouped into two: those with Tsat <20% (iron-deficient group) and those with Tsat >20% (iron-replete group). Correlations were then sought between Tsat levels in each group and the specified clinical parameter. A statistically significant weak inverse correlation was seen between age and Tsat in the iron-replete group (r* *= -0.285, p = 0.03), whereas no significant correlation existed between age and Tsat in the iron-deficient group (r* *= -0.086, p = 0.65). There was no significant correlation between Tsat and BMI, duration of HF, frequency of hospitalizations, pulse rate, systolic blood pressure and diastolic blood pressure, in both the iron-deficient and iron-replete groups (see Table 4).

**Table 4:** Correlation between selected clinical parameters and iron status, using Tsat Iron-deficient: Tsat <20% where serum ferritin <299 mcg/L; iron-replete: Tsat >20% r: Pearson’s chi-square correlation; †: Spearman’s rho correlation coefficient; BMI: body mass index; PR: pulse rate; SBP: systolic blood pressure; DBP: diastolic blood pressure; HF: heart failure; Tsat: transferrin saturation

Parameter	Iron-deficient		Iron-replete	
	r	p value	r	p* *value
Age	-0.086	0.65	-0.285	0.03
BMI	-0.230	0.22	-0.105	0.43
Duration of HF	0.300^†^	0.11	-0.214^†^	0.11
Frequency of hospitalizations	0.054^†^	0.78	0.069^†^	0.61
PR	0.071	0.71	0.010	0.94
SBP	0.014	0.94	-0.104	0.44
DBP	-0.084	0.66	-0.025	0.85

## Discussion

The clinical characteristics of the HF participants with and without ID were compared. Statistically significant differences were noted with NYHA functional classes. The ID group was mostly comprised of participants in NYHA functional classes III, 14 (46.7%), and IV, 11 (36.7%), while only five (16.7%) of the ID participants were in NYHA II. This finding is similar to previous reports by researchers. Compared with those in moderate HF (NYHA II), Jankowska et al. found that the presence of ID was significantly greater in those with advanced HF (NYHA III/IV) [23]. In the study by Klip et al., of the 753 participants with ID in chronic HF, 53% were in NYHA III, while NYHA I and II made up 39% [24]. This suggests that ID in chronic HF patients is associated with worsening HF severity, based on this functional classification. This is an expected finding as advanced HF clinically presents with impaired exercise capacity which has also been shown to bear a direct relationship with reduced tissue oxidative capacity [25]. Brutsaert et al. demonstrated muscle fatigue in non-anaemic women, which they proved was associated with reduced tissue oxidative capacity due to ID [26]. This (tissue oxidative capacity) was reported as impaired proportionally across the whole spectrum of ID (from non-anaemic to anaemic ID) [25]. Therefore, muscle fatigue, involving the myocardium and/or the skeletal muscle, may explain the worse NYHA functional classes in iron-deficient patients with chronic HF.

In this study, no correlations existed between the duration of HF and Tsat. However, a significant weak negative correlation was seen between the duration of HF and serum ferritin levels. This suggests that ID was more in participants with a longer duration of HF compared to those with a shorter duration. Jankowska et al. also reported duration of HF as an independent determinant of ID [23]. This is because long-standing inflammation has since been identified as a recipe for ID due to prolonged inhibition of intestinal absorption of iron by an IL-6 and hepcidin-mediated mechanism. No correlation was seen between the pulse rate and ferritin levels. However, a weak inverse correlation was seen between pulse rate and Tsat, which showed a signal [16]. No study has attempted to find any variation in pulse rates between iron-deficient and iron-replete individuals. However, the reason for this trend may be multifactorial. It can be extrapolated that since a higher pulse rate is an established clinical finding in anaemia (from any cause), it may be expected as ID progresses to cause anaemia. Haemodynamic responses to exertion in the iron-deficient group may also explain this finding.

This study demonstrated significantly greater proportions of participants with advanced HF (NYHA III/IV) having ID compared to those with stable HF (NYHA II). The association between ID and NYHA class cannot be over-emphasized as the latter is a demonstration of the functional capacity of these participants which may bear a direct relationship with their exercise capacity. It is expected that more persons with advanced HF would have ID compared to the less severe functional class (NYHA II). Even though advanced HF has been associated with anaemia, correction of ID, regardless of Hb status, has also been shown to significantly improve the NYHA functional class [12,27,28]. Larger interventional studies, like the FAIR-HF (Ferric carboxymaltose Assessment in patients with IRon deficiency and chronic Heart Failure) and CONFIRM-HF (Ferric CarboxymaltOse evaluatioN on perFormance in patients with IRon deficiency in coMbination with chronic Heart Failure), have also reported similar NYHA class responses to the correction of ID [29,30]. The reason for these responses may be tied to improvements in tissue oxidative capacity leading to improved exercise capacity.

Study limitations

The true prevalence of ID in HF patients may not have been obtained, as this was a hospital-based study. Also, bone marrow iron is considered a direct test for iron stores and the most reliable to evaluate iron status. Due to its invasiveness and discomfort associated with bone marrow aspiration, it was not carried out in the participants. More so, there are indirect comparable means of measuring iron status, which are readily available and have been employed in this study.

Erythrocyte ferritin is superior to serum ferritin, as it is not affected by inflammation, which is seen in HF. However, only a few machines can measure it, which are not available in our setting.

## Conclusions

There were no echocardiographic differences (using LVEF) between the ID group and participants who had no decline in their iron status. Changes in iron status were more likely to attract differences in clinical variables in chronic HF. Participants with ID showed a trend towards higher pulse rates and had advanced NYHA functional classes compared to those that were iron-replete. There was a strong association between ID and NYHA functional class: ID occurred more frequently in NYHA classes III and IV than in NYHA II. Iron studies (Tsat and serum ferritin) should be recommended for patients in NYHA III/IV who are not satisfactorily responsive to optimal heart failure therapy. Chronic HF patients with ID should be enrolled in randomized interventional trials to clarify the unmet needs of specific therapy for this patient group.
